# OCD and ASD Diagnosis: a case review

**DOI:** 10.1192/j.eurpsy.2022.991

**Published:** 2022-09-01

**Authors:** L. Huerga García, E. Hernández Padrón, N. Casanova Gracia, N. Torres Nieves, P. Gómez Pérez

**Affiliations:** 1 Hospital Universitario Nuestra Señora de La Candelaria, Psiquiatría, Santa Cruz de Tenerife, Spain; 2 Hospital Universitario Canarias, Psiquiatría, Santa Cruz de Tenerife, Spain

**Keywords:** OCD, obsessivecompulsivedisorder, acd, autismespectrumdisorder

## Abstract

**Introduction:**

It’s well known the challenge of differential diagnosis between Obssesive compulsive disorder and autism since their symptoms (intrusive, recurrent thoughts and repetitive behaviours) often overlap.

**Objectives:**

We report a case of a 14 years old boy diagnosed of ASD who was hospitalized for the first time due to difficult management of repetitive behaviours that made him incapable of doing basic activities without help. To interrupt them led to anxiety, aggressive responses and to insistence on sameness behaviours. Only with this information and with the literature research we made, anyone could tell the problem was probably an ASD symptom. However, during his evolution it was difficult to know whether this behaviour was due to ASD or OCD: after adjusting the medication, and when he started trusting his therapists, he told us about a theory he believed so he could explain the uncomfortable ideas that crossed his mind more than often, so he used those behaviours as an anxiety-reduction technique. This new situation was the fuel to make the present review.

**Methods:**

To report a case.

**Results:**

The results are included in the “conclusions” section.

**Conclusions:**

Although there is an ongoing debate concerning the nature of the symptoms in ASD versus those observed in OCD, there are commonly used criteria to differentiate them according to the articles we reviewed:

**Figure:**
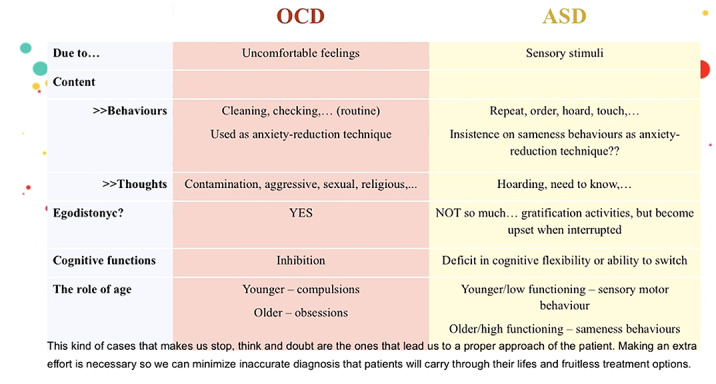

**Disclosure:**

No significant relationships.

